# Water Sorption in Glassy Polyvinylpyrrolidone-Based Polymers

**DOI:** 10.3390/membranes12040434

**Published:** 2022-04-17

**Authors:** Dominik Borrmann, Andreas Danzer, Gabriele Sadowski

**Affiliations:** Department of Chemical and Biochemical Engineering, Laboratory of Thermodynamics, TU Dortmund University, Emil-Figge-Str. 70, D-44227 Dortmund, Germany; dominik.borrmann@tu-dortmund.de (D.B.); andreas.danzer@tu-dortmund.de (A.D.)

**Keywords:** NET-GP, PC-SAFT, free volume, water sorption isotherms, water sorption kinetics

## Abstract

Polyvinylpyrrolidone (PVP)-based polymers are excellent stabilizers for food supplements and pharmaceutical ingredients. However, they are highly hygroscopic. This study measured and modeled the water-sorption isotherms and water-sorption kinetics in thin PVP and PVP-co-vinyl acetate (PVPVA) films. The water sorption was measured at 25 °C from 0 to 0.9 RH, which comprised glassy and rubbery states of the polymer-water system. The sorption behavior of glassy polymers differs from that in the rubbery state. The perturbed-chain statistical associating fluid theory (PC-SAFT) accurately describes the water-sorption isotherms for rubbery polymers, whereas it was combined with the non-equilibrium thermodynamics of glassy polymers (NET-GP) approach to describe the water-sorption in the glassy polymers. Combined NET-GP and PC-SAFT modeling showed excellent agreement with the experimental data. Furthermore, the transitions between the PC-SAFT modeling with and without NET-GP were in reasonable agreement with the glass transition of the polymer-water systems. Furthermore, we obtained Fickian water diffusion coefficients in PVP and in PVPVA from the measured water-sorption kinetics over a broad range of humidities. Maxwell-Stefan and Fickian water diffusion coefficients yielded a non-monotonous water concentration dependency that could be described using the free-volume theory combined with PC-SAFT and NET-GP for calculating the free volume.

## 1. Introduction

The production of pharmaceutical formulations and food supplements relies on polymers used as stabilizers and dispersing agents. Polyvinylpyrrolidone (PVP)-based polymers are excellent in both regards but they are also highly hygroscopic. Water sorption in these polymers induces plasticization, which aggravates the stabilizing properties of the polymers. PVP grades are usually glassy at ambient conditions in the dry state but due to their hygroscopicity quickly reach the glass transition upon water sorption. A water-containing polymer can be glassy or rubbery depending on its water uptake at specific conditions of temperature and relative humidity (RH). The equilibrium water uptake of a polymer as a function of RH is represented by its water-sorption isotherm. The curvature of the water-sorption isotherm can be either zero (linear), positive (convex), or negative (concave) with increasing RH. The shape of a sorption isotherm might show inflection points as its curvature can differ for glassy and rubbery polymers leading to distinctly differently-shaped sorption isotherms as summarized by Minelli and Sarti [1]. Most often, strictly convex or linear sorption isotherms are observed. Convex sorption isotherms are usually observed for sorption in rubbery polymers. They can successfully be described via the often applied Flory-Huggins model, as e.g., shown for the methoxyflurane sorption in silicon rubber [2]. Concave sorption isotherms are usually reported for sorption in glassy polymers [3].

Water-sorption isotherms of PVP-based polymers were frequently investigated in literature [4,5,6,7,8]. Chalykh et al. [5] measured and modeled water-sorption isotherms of PVP which could not be described using the classical Flory-Huggins approach. The parts of the water-sorption isotherm where the polymer remained glassy were concave while the parts where the polymer became rubbery were convex. Thus, the water-sorption isotherms showed an inflecting behavior with increasing RH. Similar observations were made by Davis et al. [8]. They investigated the water sorption in PVP, polystyrene, and poly(methyl methacrylate) (PMMA) and revealed insufficient capabilities of the Flory-Huggins model to describe the water-sorption isotherms of these polymers over the whole range of RH.

The Flory-Huggins model can only describe strictly convex sorption isotherms, whereas it fails to model linear or concave parts of a sorption isotherm. The reason for the differences in sorption behaviors of glassy and rubbery polymers is the lower molecular mobility in glassy polymers compared to the rubbery state which hinders reaching an equilibrium state. As a result, glassy solvent-loaded polymers are in a non-equilibrium state, and their sorption isotherms depend on their thermal history [9,10,11]. Chalykh et al. [7] found that the duration of the thermal pretreatment had a significant influence on the water-sorption isotherms of PVP samples. PVP films annealed at 160 °C for 1 h showed almost double the water uptake as those annealed for 3 h. History dependencies of the sorption isotherm suggest using a thermodynamic model capable of describing non-equilibrium states.

An early model for describing sorption in glassy polymers is the dual-mode-sorption model [1]. This model assumes a superposition of a linear sorption isotherm and a concave sorption isotherm. The central assumption here is that there are two kinds of penetrating species: one kind absorbing in the polymer and another one adsorbing on the polymer. The dual-mode-sorption model successfully describes the sorption of gases [9] or solvents [12] in glassy polymers. However, it cannot describe convex parts of a sorption isotherm. Thus, Chalykh et al. [7] applied a modification of the dual-mode-sorption model that is a superposition of the concave Langmuir sorption isotherm and the convex Flory-Huggins sorption isotherm. The superposition of the two sorption isotherms nicely reproduced the experimentally found inflecting behavior of the water-sorption isotherms of PVP mentioned earlier [5].

The thermal-history dependency of the water-sorption isotherms mentioned above was primarily observed for lower RHs and vanished for higher RHs. The modeling of Chalykh et al. [7] reflected this behavior as the Langmuir constants obtained for water-sorption isotherms of PVP samples with different thermal histories were different, while the Flory-Huggins interaction parameters χ remained almost the same. The Langmuir constant mainly influenced the modeling of the water-sorption isotherms at lower RHs where the polymer-water systems were in a non-equilibrium state, and a thermal-history dependency is reasonable. The modeling of the water-sorption isotherms at higher RHs where the polymer-water systems are rubbery and attain equilibrium was mainly influenced by the Flory-Huggins interaction parameter χ. However, superposition approaches such as the dual-mode-sorption model are limited to modeling polymer-penetrant systems [1]. Therefore, its application to multi-component systems is quite limited. Moreover, due to its empirical nature, this framework does not allow safe extrapolations to other conditions, such as temperatures or penetrant partial pressures [1].

Sarti et al. [13] developed an alternative modeling strategy, the non-equilibrium-thermodynamics of glassy polymers (NET-GP) approach, which considers the non-equilibrium nature of a glassy polymer. The NET-GP approach proposes a physically accurate representation of the glassy state when used in combination with a suitable equation of state. It was successfully applied with the equation of states PC-SAFT [14], Sanchez-Lacombe [15], and Redlich-Kwong-Soave [16]. Moreover, the NET-GP approach was successfully applied to a wide array of systems and predictions, as summarized in a review [1]. The theory behind NET-GP is based on and consistent with the polymer’s pressure-volume-temperature behavior [17].

PC-SAFT is a highly suitable equation of state with excellent predictive capabilities, especially in systems containing complex molecules and polymers [18,19]. In contrast to most other equations of state it specifically considers the association between molecules [19].

Water-sorption isotherms were first modeled using the NET-GP approach combined with the Sanchez-Lacombe equation of state by Sarti and De Angelis [20] for describing the water sorption in polycarbonate. Sorption of water and of ethanol over a broad range of temperatures from 25 to 125 °C was described using the same set of model parameters for polycarbonate. A recent study by Lui and Kentish [21] investigated the non-equilibrium water sorption in polylactide (PLA) and PMMA. By combining PC-SAFT and NET-GP, they apparently found only a slight difference between the PC-SAFT modeling with or without NET-GP (using different binary interaction parameters between water and the polymer for the two cases). However, due to very small water uptakes, both PLA and PMMA-water systems were exclusively glassy over the whole range of RHs. Therefore, differences between the water sorption behavior of glassy and rubbery polymer-water mixtures did not occur in these cases. However, although the modeling of glassy polymer-water systems was possible without NET-GP, it is questionable that the fitted binary interaction parameter could also be used for extrapolations to the rubbery state.

In contrast, the modeling of the water-sorption isotherms of PVP-based polymers is quite different, as PVP does not remain only glassy or rubbery over the considered RH range. In this work, we used PC-SAFT to model the water-sorption isotherms of rubbery PVP and combined it with the NET-GP approach to feature a good description of the water-sorption isotherms in the glassy PVP.

Besides the water-sorption isotherms, some authors [5,6] also investigated water-sorption kinetics using polymer films instead of powders. For example, Chalykh et al. [5] determined water-sorption kinetics in PVP films, which showed exclusively Fickian characteristics in integral sorption runs. However, the Fickian water diffusion coefficients below the glass transition decreased with increasing water concentration, whereas above the glass transition, the Fickian water diffusion coefficients decreased with decreasing water concentration. Chalykh et al. [5] explained the decreasing water diffusion coefficients below glass transition by assuming a decrease of free volume in the polymer likely caused by its plasticization upon water uptake. Nuclear magnetic resonance spectroscopy measurements from Oksanen and Zografi [6] in PVP-water systems above the glass transition found increasing Fickian water diffusion coefficients at increasing water concentration.

It is well known that the concentration dependency of the Stefan Maxwell diffusion coefficient is much more reasonable than that of the Fickian diffusion coefficient as it is based on chemical-potential gradients rather than on concentration gradients [22]. However, the calculation of the Maxwell-Stefan water diffusion coefficient requires modeling the chemical potential of water using a suitable thermodynamic model.

To conclude, the water uptake of PVP-based polymers is significant and causes the transition from the glassy state to the rubbery state of the polymer. Therefore in this work, the complex shape of the water-sorption isotherms of such polymers was modeled via combining PC-SAFT and NET-GP. Moreover, the influence of changing from the glassy to the rubbery state on the water-sorption kinetics and the water diffusion coefficient’s dependency on water concentration was investigated.

## 2. Materials and Methods

### 2.1. Materials

Polyvinylpyrrolidone (PVP) [CAS Nr. 9003-39-8] ($M_{p}=25,700\;g/mol$, grade K25) was purchased from Sigma-Aldrich and used as received. The copolymer PVPVA [CAS Nr. 25086-89-9] ($M_{p}=65,000\;g/mol$, grade VA64) was purchased from Dow Chemicals. Ethanol with a purity greater than 99.9% (LiChroSolv) was purchased from Merck.

### 2.2. Film Preparation

Thin films of PVP and PVPVA were prepared by coating a circular coverslip (18 mm in diameter) with a polymer solution using a spin coating device (Süss MicroTec D80T2 spin coater). First, the coverslips were cleaned in an ethanol bath by applying ultrasonic sound for 5 min. Then, a 14 mm diameter hole was punched out of an adhesive polystyrene foil and cut into a strip. This polystyrene strip was used to mask the coverslip. Next, 600 µL of an ethanolic polymer solution (ethanol weight fraction 0.5 for PVPVA films and ethanol weight fraction 0.6 for PVP films) was suspended on the masked coverslip. The rotating disk of the spin coater was immediately accelerated to 100 rpm/s to reach a rotational speed of 1500 rpm for PVPVA films and 1200 rpm for PVP films for 180 s. After the coating, the mask was peeled off, revealing a film approximately the size of the hole punched into the polystyrene strip. Finally, the films were annealed at 180 °C on a hot plate for 10 min and then removed from the hot plate. Before and after the coating, the coverslips were weighed using a fine balance (Mettler Toledo) with 0.1 mg of precision to determine the mass of the polymer film.

### 2.3. Quality of the Films

**Figure 1:**
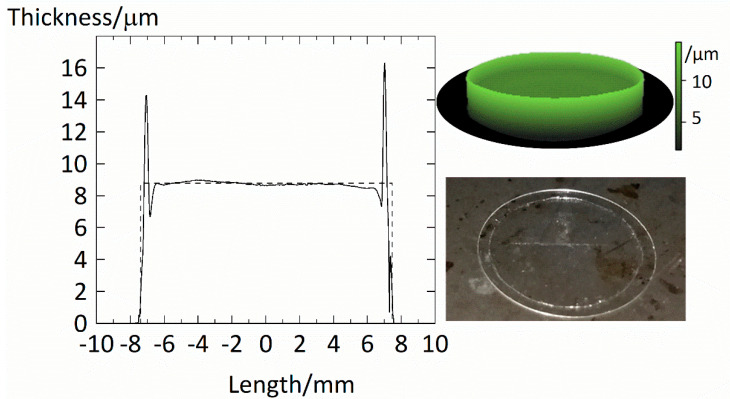
Thickness profile of PVPVA film fixed on a coverslip. The solid line indicates the measurement along the film’s length. In contrast, the dashed line represents the estimated thickness calculated using the film’s diameter, mass, and density of amorphous PVPVA of 1190 kg/m^3^. We further provide a 3D film model in the upper right corner, where a brighter tone corresponds to a higher thickness.

We used a Dektak V200 SI by Veeco to scan the surface of the films. A diamond stylus (25 µm in radius) caught the thickness profile of the films. The thickness profile of an example PVPVA film is shown in Figure 1.

Overall, the film spans over a diameter of d=14.5 mm. The film shows an almost constant thickness of ~9 µm. The increased borders of ~15 µm at the edge resulted from the peeling of the masking as the polystyrene strip was significantly thicker than the actual film. However, this border is skinny, and its contribution to the overall volume of the film is negligible. We concluded that the films prepared by the spin-coating method produce very even films with almost constant thicknesses. The thickness $L_{0}=\frac{4m_{0}}{\rho_{0p}\pi d^{2}}$ of the dry polymer film was calculated using reported film densities $\rho_{0p}$ of the polymer and the mass of the dry polymer film $m_{0}$. With a value of $L_{0}=8.78\;µm$, the estimated average thicknesses of this film matches very well the average thickness of 8.9 µm experimentally determined from the thickness profile.

### 2.4. Water-Sorption Measurements

Water sorption of the PVP and PVPVA films was determined at 25 °C via a DVS Intrinsic Plus System (0.1 µg) from Surface Measurements Systems. The samples were dried in the measurement cell before the experiment at an RH of 10^−5^ for at least 12 h. The total mass after the drying step represents the dry mass $m_{0}$ of the polymer. Successive step-wise changes in RH were investigated. The duration of each RH step was 200 min for RH steps below 0.6 and 120 min for higher RHs. These durations were determined during a preliminary test run and resulted in sorption rates at the end of the sorption step that were lower than 0.212 µg/g/min (as shown in the Supplementary Material in Table S1). The mass of water $m_{w}$ was determined as the difference between the readings of the total mass m at any time and the dry mass $m_{0}$ of the polymer. The water weight fraction $w_{w}$ was calculated as the ratio of the mass of water $m_{w}$ and the total mass m. Every sorption measurement was a triple determination and the average values are reported.

## 3. Theory

### 3.1. PC-SAFT

The perturbed-chain statistical associating fluid theory (PC-SAFT) provides an expression for the reduced residual Helmholtz energy $a^{res}$ which is displayed in Equation (1).

(1) $$a^{res}=a^{hc}+a^{disp}+a^{assoc}\;$$

$a^{hc}$ is the reduced Helmholtz Energy contribution for the hard-chain reference fluid. $a^{disp}$ is the dispersion contribution accounting for van der Waals attractions and $a^{assoc}$ the association contribution. The expressions for the terms $a^{hc},a^{disp},a^{assoc}$ can be found elsewhere [18,19]. These expressions use the parameters of the PC-SAFT model: the segment diameter $\sigma_{i}$, the segment number $m_{i}$, the association volume $\kappa^{AiBi}$, the dispersion energy parameter $u_{i}/k_{B}$ and the association energy parameter $\varepsilon^{AiBi}/k_{B}$ of component i where $k_{B}$ is the Boltzmann constant. We applied Berthelot-Lorenz mixing rules to calculate the mixture’s segment diameter $\sigma_{ij}\;$, and the mixture’s dispersion energy $u_{ij}$. Wolbach and Sandler [23] mixing rules were used to calculate the cross-association energy $\varepsilon^{AiBj}$ and the cross-association volume $\kappa^{AiBj}$. We summarize the applied mixing rules in Table 1. The binary interaction parameter $k_{ij}$ corrects deviations of the mixture’s dispersion energy $u_{ij}$ from the geometric mean of the pure-component values.

Considering the polymer-water systems in this work, the mixture consists of component i,j∈{w,p} with water w and polymer p. Thus, the fugacity $f_{i}$ of component i was calculated according to Equation (2).

(2) $$ln\left(f_{i}\right)=a^{res}+{\left(\frac{\partial a^{res}}{\partial x_{i}}\right)}_{T,\tilde{\rho },x_{j\neq i}}-\underset{j}{\sum }x_{j}{\left(\frac{\partial a^{res}}{\partial x_{j}}\right)}_{T,\tilde{\rho },x_{j\neq i}}+Z-1+ln\left(\tilde{\rho }x_{i}k_{B}T\right)\;$$

Here,$\;\tilde{\rho }$ is the number density and Z is the compressibility factor which is calculated according to Equation (3).

(3) $$Z=1+\tilde{\rho }{\left(\frac{\partial a^{res}}{\partial \tilde{\rho }}\right)}_{T,x_{i}}\;$$

### 3.2. NET-GP

The pressure-volume-temperature (PVT) behavior of the glassy polymer differs substantially from its rubbery counterpart. These differences arise from low molecular mobility in the glassy polymer leading to a prolonged time-dependent relaxation. Given the time frames of penetrant diffusion (here, water is the penetrant), the glassy polymer reaches a nearly constant yet non-equilibrium volume. Due to the altered PVT behavior of the glassy polymer, the volume $V^{NE}$ in this non-equilibrium state (NE) is different from the volume $V^{EQ}$ in the equilibrium state (EQ). Naturally, the inequality of the two volumes $V^{NE}\neq V^{EQ}$ is the property characterizing the non-equilibrium state. Therefore, Sarti and Doghieri [24] introduced the volume $V^{NE}$ in the non-equilibrium state as an additional state variable. They showed that any function that calculates a reduced Helmholtz energy $a^{EQ}$ for the equilibrium state also calculates a valid reduced Helmholtz energy $a^{NE}$ for the non-equilibrium state and Equation (4) holds. (More details are can be found in the original sources [24,25]).

(4) $$a^{NE}\left(T,p,V^{NE},x_{i}\right)=a^{EQ}\left(T,V^{NE},x_{i}\right)\;$$

The reduced Helmholtz energy $a^{NE}$ for the non-equilibrium state at a certain pressure p and temperature T corresponds to the reduced Helmholtz energy $a^{EQ}$ for the equilibrium state at the same temperature but with the volume $V^{NE}$ in non-equilibrium. As a result, the volume $V^{NE}$ in non-equilibrium is obtained from the reduced Helmholtz energy function and not calculated from the system pressure p. In systems with a significant amount of penetrant uptake, the volume $V^{NE}$ in non-equilibrium is a function of the penetrant partial pressure [1]. In this work, we assumed that the volume dilation, i.e., the ratio of the volume $V^{NE}$ in non-equilibrium at a particular water concentration and the dry volume $V_{0}^{NE}$ in non-equilibrium is a quadratic function of the relative humidity RH (Equation (5)).

(5) $$\frac{V_{0}^{NE}}{V^{NE}}=\frac{v_{0p}^{NE}}{v^{NE}}w_{p}=1-k_{wp}^{NE}RH^{2}\;$$

Here, $RH=\frac{p_{w}}{p_{0w}}$ is the ratio of the water partial pressure $p_{w}$ and the water vapor pressure $p_{0w}$. Equation (5) introduces two adjustable parameters: $k_{wp}^{NE}$ which is the swelling coefficient of the polymer by water and $v_{0p}^{NE}$ which is the specific volume of the dry polymer in non-equilibrium.

### 3.3. Water-Sorption Isotherm

The thermodynamic equilibrium condition for the rubbery polymer-water system requires the equality of the fugacities of water in both liquid L and vapor V phase (Equation (6)).

(6) $$f_{w}^{L}\left(T,p,V^{EQ}\left(T,p\right),x_{i}\right)=f_{w}^{V}\left(T,p\right)\;$$

Here, $f_{w}^{L}$ is the fugacity of water in the liquid phase whereas $f_{w}^{V}$ is the one in the vapor phase. The fugacity $f_{w}^{L}$ is evaluated at the volume $V^{EQ}$ in the equilibrium state which corresponds to systems temperature T and pressure p. The vapor phase consists of pure water vapor at the system pressure p which is then equal to the partial pressure $p_{w}$ of water.

In contrast, as the volume relaxation of the glassy polymer-water system occurs in time frames much longer than the experimental ones (i.e $\frac{\partial V^{NE}}{\partial t}\approx 0$), the glassy system is presumed to be in a pseudo-equilibrium with the vapor phase [24]. This way, a pseudo-equilibrium condition, analogously to Equation (6), follows as displayed in Equation (7).

(7) $$f_{w}^{L}\left(T,p,V^{NE},x_{i}\right)=f_{w}^{V}\left(T,p\right)$$

Here, the fugacity $f_{w}^{L}$ of water in the liquid phase is evaluated at the volume $V^{NE}$ in non-equilibrium, which is calculated from Equation (5). As the polymers considered in this study cross the glass transition when reaching specific RHs, the fugacity $f_{w}^{L}$ of water in the liquid phase changes from the non-equilibrium value to the equilibrium one and a transition relation must be formulated. The glass transition is a phase transition of second-order and first derivatives of the Helmholtz energy with respect to the state variables are continuous at its transition. As a result, the transition relation that was used in this work is presented in Equation (8).

(8) $$f_{w}^{L}=\{\begin{array}{c} f_{w}^{L}\left(T,p,V^{EQ}\left(T,p\right),x_{i}\right)\;\;\;\;\;\;\;\;\;\;\;if\;x_{w}^{EQ}>x_{w}^{NE}\\ f_{w}^{L}\left(T,p,V^{NE},x_{i}\right)\;\;\;\;\;\;\;\;\;\;\;\;\;\;\;\;\;\;\;\;\;if\;x_{w}^{EQ}\leq x_{w}^{NE} \end{array}\;$$

Here, $x_{w}^{EQ}$ is the water mole fraction that fulfills the equilibrium condition (Equation (6)) and $x_{w}^{NE}$ is the water mole fraction that fulfills the pseudo-equilibrium condition (Equation (7)). Thus, the transition requirement that is imposed by Equation (8) is $x_{w}^{EQ}=x_{w}^{NE}$ which also implies $x_{p}^{EQ}=x_{p}^{NE}$. At that point, also the volume in non-equilibrium $V^{NE}$ is the same as the volume $V^{EQ}$ in equilibrium. Hence, the fugacity of water $f_{w}^{L}$ in the liquid phase is a continuous function at the transition. Both equilibrium and pseudo-equilibrium calculations were performed for the full range of RHs from zero to one as it is not clear a priori whether and at which RH the condition $x_{w}^{EQ}=x_{w}^{NE}$ is satisfied. The transition point then results as the intersection point of the two water-sorption isotherms obtained from Equations (6) and (7).

### 3.4. Model Parameters

The pure component PC-SAFT parameters for PVP, PVPVA, polyvinyl acetate (PVAc), and water were taken from previous works [26,27,28,29]. The PC-SAFT parameters and NET-GP parameters that were used in this study are listed in Table 2.

The non-equilibrium parameters $v_{0p}^{NE}$ and $k_{wp}^{NE}$ were fitted to the parts of the water-sorption isotherms where the polymer-water mixture was glassy. The binary interaction parameters $k_{wp}$ between polymer and water were exclusively obtained from the parts of the water-sorption isotherms at high RHs where the polymer-water mixture was rubbery.

### 3.5. Water-Sorption Kinetics

The solution of Fick’s second law for the diffusion in a thin film of thickness $L_{0}$ was given by Cranc [31]. Thus, we calculated the mass of water in the film $m_{w}$ using Equation (9).

(9) $$m_{w}=\left(m_{w}^{\infty }-m_{w}^{0}\right)\left(\;1-\overset{\infty }{\underset{q=0}{\sum }}\frac{8}{\pi^{2}{\left(2+q\right)}^{2}}\exp\left({\left(2+q\right)}^{2}\frac{D_{wp}}{4L_{0}^{2}}\;\;t\right)\right)+m_{w}^{0}\;$$

Here, $m_{w}^{\infty }$ is the mass of water in at the end of the sorption step and $m_{w}^{0}$ is the water mass already present at the start of the sorption step. Thus, both of these water masses correspond to the water weight fraction $w_{w}^{\infty }$ at the end of the sorption step and $w_{w}^{0}$ at the start of the sorption step, which are directly related to the water-sorption isotherm. q is an index used for approximating the infinite sum (here 20 summands were sufficient) and t is the time.

Polyvinyl-based polymers absorb large amounts of water. Due to this, the thickness L of the film strongly increases during sorption. The increasing difference between the time-depended thickness of the film L and the thickness of the dry film $L_{0}$ increases the effective diffusion pathway, which in turn reduces the mass flux. Cranc [31] showed that the volume expansion during sorption manifests itself in a scalar prefactor. We considered this volume expansion by the factor $\omega_{0}^{2}={\left(\frac{L_{0}\;A}{L\;A}\right)}^{2}$ assuming that the cross-sectional area A of the film does not change during sorption. The Fickian diffusion coefficient $D_{wp}$ of water in the polymer was obtained from fitting to Equation (9) the water-sorption curves. The fitting (by SciPy’s [32] Levenberg-Marquardt algorithm) minimizes the summed square of deviations of measured and modeled water sorption data.

### 3.6. Maxwell-Stefan Diffusion

We used the Maxwell-Stefan formalism to calculate Maxwell-Stefan diffusion coefficients for the diffusion modeling [22]. Equation (10) describes the diffusion of a component i in a thin film [33].

(10) $$\frac{\partial \rho_{i}}{\partial t}=\frac{\partial }{\partial z}\left(\rho_{i}\frac{{Đ}_{ij}}{x_{j}}\;{\left(\frac{\partial lnf_{i}^{L}}{\partial z}\right)}_{T}\right)\;\;$$

Here, z is the spatial coordinate perpendicular to the surface of a polymer film of thickness L. The quantity $\rho_{i}=\frac{m_{i}}{V}$ represents the concentration of component i, where $m_{i}$ is the mass of component i and V is the volume of the film. ${Đ}_{ij}$ is the Maxwell-Stefan diffusion coefficient of component i in the component j and $x_{j}$ is the mole fraction of component j. The chemical-potential gradient was expressed by the fugacity gradient $\frac{\partial lnf_{i}^{L}}{\partial z}$ of component i. The fugacity gradient $\frac{\partial lnf_{i}^{L}}{\partial z}$ is obtained from the mole fraction gradient $\frac{\partial lnx_{i}}{\partial z}$ times the thermodynamic factor $\Gamma_{i}$ of component i. This way, Equation (11) relates the Fickian diffusion coefficient $D_{ij}$ and the Maxwell-Stefan diffusion coefficient ${Ð}_{ij}$ component i in component j.

(11) $$D_{ij}={Ð}_{ij}{\left(\frac{\partial lnf_{i}^{L}}{\partial lnx_{i}}\right)}_{T}={Ð}_{ij}\Gamma_{i}\;$$

As molar masses of polymers and solvents are quite different, resulting in very low polymer mole fractions, we modified Equation 11 using the segment-molar Maxwell-Stefan formalism proposed by Fornasiero et al. [34]. We compared the original formulation [22] and the one presented by Fornasiero et al. [34] and obtained the relation for the Maxwell-Stefan diffusion coefficients in both formalisms as seen in Equation 12.

(12) $${Đ}_{ij}={Đ}_{ij}^{\prime \prime }r_{i}\frac{x_{j}}{w_{j}}\;\;\;$$

Here, $r_{i}$ is the segment number of component i.${\;Đ}_{ij}^{\prime \prime }$ is the segmental Maxwell-Stefan diffusion coefficient between a segment of component i and a segment of component j. In this work, the mass of a water molecule defines a segment, whereas the segment number $r_{i}=\frac{M_{i}}{M_{w}}$ of component i relates to the ratio of its molar mass $M_{i}$ to the molar mass of water $M_{w}$. The Maxwell-Stefan diffusion coefficients ${Đ}_{ij}^{\prime \prime }$ of the segments retain their symmetric properties just as ${Đ}_{ij}$ and therefore fulfill Onsager’s reciprocal relations [34]. This means that the two Maxwell-Stefan diffusion coefficients of a component pair i and j are identical (${Đ}_{ij}^{\prime \prime }={Đ}_{ji}^{\prime \prime }$).

Inserting Equation (12) into Equation (11) one obtains Equation 13 after some rearrangements.

(13) $$D_{ij}={Đ}_{ij}^{\prime \prime }\frac{\partial lnf_{i}^{L}}{\partial lnx_{i}}r_{i}\frac{x_{j}}{w_{j}}={Đ}_{ij}^{\prime \prime }\frac{\partial lnf_{i}^{L}}{\partial lnw_{i}}r_{i}={Đ}_{ij}^{\prime \prime }\Gamma_{i}^{\prime \prime }\;$$

Since it can be shown that $\frac{x_{j}}{w_{j}}=\frac{\partial \ln\left(x_{i}\right)}{\partial \ln\left(w_{i}\right)}$, the prefactor now is $\Gamma_{i}^{\prime \prime }=\frac{\partial lnf_{i}^{L}}{\partial ln\left(w_{i}\right)}r_{i}$. Thus, the thermodynamic factor $\Gamma_{i}^{\prime \prime }$ of a segment of component i is an analogue to the thermodynamic factor $\Gamma_{i}$ and corrects for the non-idealities between a segment of component i and a segment of component j.

The Maxwell-Stefan diffusion coefficient ${Đ}_{wp}^{\prime \prime }$ of water in the polymer was then calculated according to Equation (14).

(14) $${Đ}_{wp}^{\prime \prime }=\frac{D_{wp}}{\omega_{0}^{2}\Gamma_{w}^{\prime \prime }}\;$$

PC-SAFT and NET-GP were used to calculate the thermodynamic factor $\Gamma_{w}^{\prime \prime }$ of water to account for non-idealities. The thickness $L_{0}$ of a dry film was approximated using the mass and the pure densities $\rho_{oi}$ of dry PVP and PVPVA from Table 2. The thickness L was calculated by assuming volume additivity and using the density of pure water. Since both $\omega_{0}^{2}$ and $\Gamma_{w}^{\prime \prime }$ depend on the water concentration, these quantities were evaluated at an intermediate value of the water weight fraction $0.3w_{w}^{0}+0.7w_{w}^{\infty }$ for each sorption step. The change of $\omega_{0}^{2}\Gamma_{w}^{\prime \prime }$ during a sorption step was small, justifying the use of an average of $\omega_{0}^{2}\Gamma_{w}^{\prime \prime }$ for the determination of ${Đ}_{wp}^{\prime \prime }$. The thermodynamic factors $\Gamma_{w}^{\prime \prime }$ were calculated using the transition rule of $f_{w}^{L}$ in Equation (8), hence featuring PC-SAFT modeling with and without NET-GP.

### 3.7. Free-Volume Theory

The free volume theory [35] was used to model the water concentration dependency of the Maxwell-Stefan diffusion coefficient ${Đ}_{wp}^{\prime \prime }$ of water in the polymer via the free volume $V_{f}$ which is the volume not occupied by the volume $V_{occ}$ of the molecules. For this purpose, an empirical relation in Equation (16) (which is based on the free volume theory [36]), was used to describe the intradiffusion coefficient $D_{wp}^{FV}$ of the water in the polymer. Consequently, the intradiffusion coefficient $D_{wp}^{FV}$ of water in the polymer relates to the corresponding segmental Maxwell-Stefan diffusion coefficient ${Ð}_{wp}^{\prime \prime }$ in Equation (15).

(15) $$D_{wp}^{FV}=\frac{{Ð}_{wp}^{\prime \prime }}{1-w_{w}}=D_{0w}\;exp\left(-\frac{B}{FFV}\right)\;$$

Here, the prefactor $D_{0w}=8.55\cdot 10^{-8}\;\frac{m^{2}}{s}$ for water is independent of the polymer and was taken from the literature [37]. The constant B was fitted to the water concentration dependency of ${Ð}_{wp}^{\prime \prime }$. The fractional free volume FFV is the ratio of free volume $V_{f}$ and the volume V of the system and is calculated according to Equation (16).

(16) $$FFV=\frac{V_{f}}{V}=\frac{V-V_{occ}}{V}=1-\frac{v_{occ}}{v}=1-\frac{v_{0w}^{hc}w_{w}+v_{0p}^{hc}w_{p}\zeta }{v}\;$$

The specific volume $v_{occ}$ of the molecules was approximated by calculating the specific volume $v_{0i}^{hc}$ of the hard-chains of each component i according to $v_{0i}^{hc}=\frac{\pi }{6}\sigma_{i}^{3}\frac{m_{i}}{M_{i}}N_{av}\;$, where $N_{av}$ is the Avogadro’s constant. The specific volume v of the mixture was calculated using PC-SAFT with and without NET-GP. The transition between the two *FFV* descriptions was performed using the same criterium as for the transition between the water sorption isotherms in Equation (8). The volumetric ratio ζ of the jumping units of water and the polymer was fitted to the water concentration dependency of ${Ð}_{wp}^{\prime \prime }$

## 4. Results

### 4.1. Water-Sorption Isotherms

**Figure 2:**
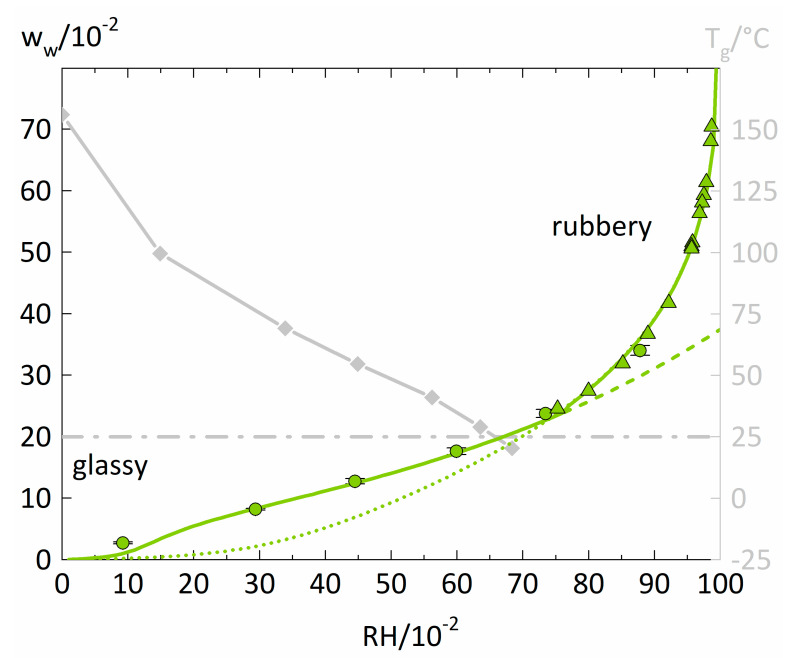
Water-sorption isotherm of PVP at 25 °C (left y-axis) and glass-transition temperatures of PVP-water mixtures (right y-axis). Water sorption measured in this study is displayed as circles. The PC-SAFT modeling without NET-GP is presented as a dotted line. The PC-SAFT modeling with NET-GP is shown as a dashed line. The combination of NET-GP and PC-SAFT is displayed as a thick solid line. Additionally, isopiestic measurements of PVP-water solutions in the high-humidity range were taken from the literature [39] and are displayed as triangles. The PVP-water mixture’s T_g_ values were taken from the literature [38] (diamonds). The water concentration resulting in a T_g_ of 25 °C is displayed as a dash-dotted horizontal line.

Measured and modeled isotherms for water sorption in PVP are displayed in Figure 2 together with the PVP-water mixture’s glass transition temperatures T_g_ from the literature [38] featuring the same grade as our PVP (PVP K25 M_w_ = 25,700g/mol).

The water-sorption isotherm of PVP increases almost linearly until RH = 0.6. After 0.6 RH, there is a slight upward curvature of the water-sorption isotherm. Isopiestic measurements of PVP-water solutions from the literature [39] fit very well into the trend of our sorption isotherms. The sharp increase of water uptake results from the complete miscibility of PVP and water and smooth conversion from a glassy polymer film into a liquid PVP-water solution. The PC-SAFT modeling of the water-sorption isotherm shows an excellent agreement for high RHs, especially in describing the isopiestic measurements of PVP-water solutions taken from the literature [39]. For RH values smaller than 0.6, one observes a significant underestimation of the water sorption by the PC-SAFT modeling. This is because PC-SAFT alone does not consider the polymer-water mixture’s changing PVT behavior below the glass transition. In contrast, the NET-GP modeling performs excellent below 0.6 RH. However, it fails to describe the rubbery polymer-water mixtures where the PC-SAFT modeling alone gives accurate descriptions. The transition between the two modeling approaches manifests itself in an intersection point of the PC-SAFT modelings with and without NET-GP and the combination of both modeling approaches features an excellent representation of the overall water-sorption isotherm.

An RH of ~0.63 leads to a water concentration in the polymer that results in a T_g_ of 25 °C and represents the glass transition of the PVP-water mixture at the measurement temperature. This point is in very good agreement with the intersection point of the two model approaches located at ~0.73 RH. Thus, the transition of PC-SAFT with and without NET-GP is a decent approximation for the glass transition of polymer-water mixtures.

**Figure 3:**
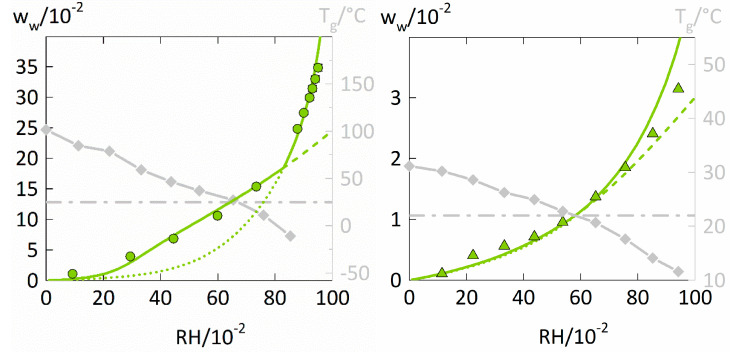
Water-sorption isotherms at 25 °C in PVPVA and PVAc (left y-axis) as well as the T_g_s of PVPVA-water and PVAc-water mixtures(right y-axis). The water-sorption isotherm of PVPVA (left diagram) measured in this study is displayed as circles. The water-sorption isotherm in PVAc (right diagram) was taken from the literature [40] and is displayed as triangles. The T_g_s of PVPVA-water and PVAc-water mixtures were also taken from the literature [40] (diamonds). The water concentration resulting in a T_g_ of 25 °C is displayed as a dash-dotted horizontal line. PC-SAFT modeling without NET-GP is presented as dotted lines, PC-SAFT modeling with NET-GP is presented as a dashed line and the combined approach is displayed as a thick solid line.

The water-sorption isotherms of PVPVA are displayed in Figure 3 on the left. Additionally, the water-sorption isotherms of a low-water-absorbing polymer, here PVAc on the right of Figure 3, were taken from the literature [40] and modeled with PC-SAFT and NET-GP to investigate the influence of the total water uptake on the performance of the two modeling approaches.

The water-sorption isotherm of PVPVA shows a strictly convex curvature and the water sorption at high RHs is satisfactorily described by the PC-SAFT modeling without NET-GP. In contrast, the NET-GP modeling much better describes the parts of the water-sorption isotherm for the glassy polymer-water mixture. For PVAc (Figure 3 on the right), the PC-SAFT modeling without NET-GP accurately describes the whole water-sorption isotherm. For RHs below 0.6, the PC-SAFT modeling with NET-GP further is almost identical to the PC-SAFT modeling without NET-GP. Thus, the influence of the NET-GP approach considerably differs for modeling the water sorption in PVAc compared to the one in PVP and PVPVA.

The glass transition of PVAc-water mixtures is crossed at a similar RH of 0.6 as for the PVP-water and PVPVA-water mixtures, which suggests a similar change in the PVT behavior for the three polymers. However, PVAc absorbs significantly less water than PVP and PVPVA with at most ~0.03 water weight fraction at 0.95 RH. Lui and Kentish [21] also reported only small differences between PC-SAFT modelings with and without NET-GP when considering water-sorption isotherms of low-water-absorbing polymers. They investigated the polymers PLA and PMMA which absorbed even less water than PVAc, with at most 0.015 water weight fraction for PMMA at 0.9 RH. However, both systems did not cross the glass transition over the whole range of considered RHs.

As a result, the improvement achieved by considering the NET-GP approach does not mainly depend on the fact whether or not the polymer-water mixture crosses the glass transition but likely depends on the total water uptake of the polymer. For describing water-sorption isotherms of low-water-absorbing polymers, considering hydrogen bonding by PC-SAFT seems to be more important than reproducing the accurate PVT behavior. Thus, applying the NET-GP approach is certainly more important for polymers that absorb high amounts of water.

### 4.2. Water-Sorption Kinetics

**Figure 4:**
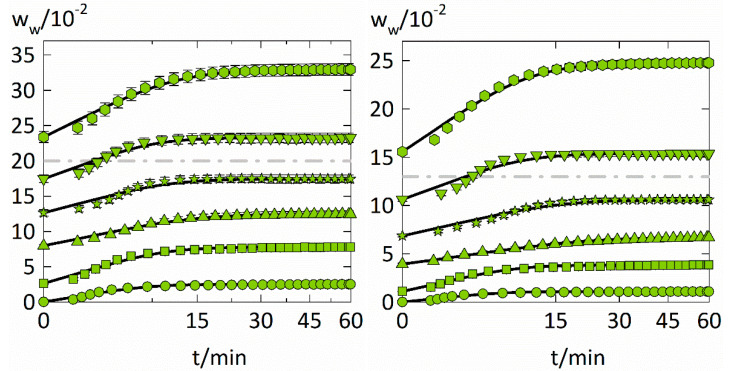
Evolution of the water weight fraction in the investigated polymer films (PVP on the left and PVPVA on the right) at six different RH step changes at T = 25 °C against the square root of time. Each of the step changes is displayed with a different symbol (circles: 0 to 0.1 RH, squares: 0.1 to 0.3 RH, upside triangles: 0.3 to 0.45 RH, stars: 0.45 to 0.6 RH, downside triangles: 0.6 to 0.75 RH, hexagons: 0.75 to 0.9 RH), while the descriptions using the Fickian diffusion model are indicated as solid lines. In addition, the glass transitions taken from the literature [38,40] are displayed as dash-dotted lines for PVP and PVPVA.

**Figure 5:**
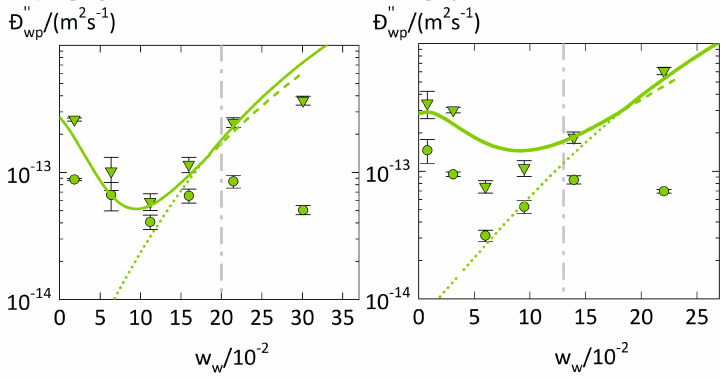
Fickian diffusion coefficients $D_{wp}$ (circles) and Maxwell-Stefan diffusion coefficients ${Đ}_{wp}^{\prime \prime }$. (triangles) of water in polymers at T = 25 °C as function of the average water weight fraction ($0.7w_{w}^{\infty }+0.3w_{w}^{0})$. of a sorption step. The left diagram displays water diffusion coefficients in PVP, and the right diagram those in PVPVA. The glass transitions are indicated as dashed lines as derived from literatur [38,40].he modeling results of ${Đ}_{wp}^{\prime \prime }$. obtained from Equation (15) using PC-SAFT without NET-GP are presented as dotted lines, PC-SAFT modeling results with NET-GP are presented as dashed lines, the combined approach is displayed as thick solid line.

The experimental data of the experimental water-sorption kinetics is displayed together with a fitting (Equation (9)) in Figure 4. Obtained Fickian water diffusion coefficients in PVP and PVPVA for each step change in RH are shown in Figure 5 and Table 3. Fickian sorption behavior would appear in Figure 4 as a linear course of the sorption curves for the first 60% of total water uptake. However, slow volume relaxation of the polymer influences water diffusion and leads to sorption behaviors that deviate from Fick’s laws, so-called anomalous sorption behavior as frequently observed for solvent sorption in polymers [41].

The water-sorption kinetics in the two polymers behave quite similarly despite the different degrees of water uptake. The water-sorption kinetics are mostly sigmoidal for both polymers and the RH steps 0.45–0.6 and 0.6–0.75 (in the vicinity of the glass transition) show the strongest sigmoidal characteristics, meaning that the experimental data deviate from the Fickian model at short times.

Above 0.75 RH, almost Fickian-like behavior is observed for both polymers. This can be explained by the fact that the polymer-water mixtures become more liquid-like above the glass transition. This speeds up molecular mobility and leads to a smaller influence of volume relaxation on sorption kinetics. Therefore, the sorption curves for higher RHs behave less anomalous and faster reach equilibrium. This also explains why the PC-SAFT modeling alone (without NET-GP) succeeds in modeling the water-sorption isotherms at high RHs.

Sorption curves below 0.45 RH, which are far below the glass transition, appear less anomalous than those in the vicinity of the glass transition. These sorption curves behave pseudo-Fickian [42], meaning that they look Fickian-like but are in “frozen” a pseudo equilibrium. For that reason, they were only reproducible when using PC-SAFT together with NET-GP.

Fitting the second Fick’s law to the data from Figure 4 led to water diffusion coefficients listed in Table 3. The Maxwell-Stefan diffusion coefficients ${Đ}_{wp}^{\prime \prime }$ were obtained through Equation (14). As anomalous sorption behavior was experimentally observed but not considered during the modeling of a sorption step, the Fickian and Maxwell-Stefan diffusion coefficients inherit the effects of the glassy polymer’s slow volume relaxation. This is also reflected in the average relative deviations of the fittings which are the greatest for the lowest RH step (~10%) but decrease to about 0.5 % for the highest RH step. (listed in the Supplementary Material in Table S1). As a result, these water diffusion coefficients might not have physical meaning since the water diffusion was not only controlled by diffusion but also volume relaxation. This fact limits the comparability of these results to the water diffusion coefficients in these polymers obtained by modeling approaches that consider volume relaxation during solvent diffusion in polymers as the one developed in our previous study [43].

### 4.3. Concentration Dependency of the Water Diffusion Coefficient

The diffusion coefficients ${Đ}_{wp}^{\prime \prime }$. and $D_{wp}$. from Table 3 are shown in Figure 5 as a function of the water weight fraction in the polymer together with the modeling of ${Đ}_{wp}^{\prime \prime }$. using Equation (15). We obtained the constants B=3.9 for PVP and B=3.5. for PVPVA. The jumping unit was found to be ζ=1.8. for both polymers.

The water diffusion coefficients ${Đ}_{wp}^{\prime \prime }$. and $D_{wp}$ in the two polymers show very similar and non-monotonous water concentration dependencies. They first decrease with increasing water weight fraction, reach minima for water weight fractions of 0.11 for PVP and 0.06 for PVPVA and then rise again. The water concentration dependency of ${Đ}_{wp}^{\prime \prime }$ obviously is more reasonable than the one of $D_{wp}$. since it shows only one trend change which moreover qualitatively follows the free-volume theory.

The minima in the water concentration dependencies of ${Đ}_{wp}^{\prime \prime }$ occur at water weight fractions lower than the glass transitions (at ~0.2 for PVP and ~0.13 for PVPVA). Thus, there obviously happens an essential change in the diffusion behavior in the polymer-water mixture even below the glass transition. According to the free-volume theory (Equation (15)), ${Đ}_{wp}^{\prime \prime }$ is determined by the fractional free volume FFV. Water has a bigger free volume than most polymers as it is well above its glass transition [37]. Increasing the water weight fraction in the polymer leads to an increasing contribution of water to FFV. explaining the increase in ${Đ}_{wp}^{\prime \prime }$ with increasing the water weight fraction. However, the non-monotonous water concentration dependency of ${Đ}_{wp}^{\prime \prime }$. suggests the existence of an additional, opposite effect that decreases the FFV upon increasing the water weight fraction. Since FFV depends on the specific volume *v* of the polymer-water mixture (Equation (16)), the water concentration dependency of ${Đ}_{wp}^{\prime \prime }$ is mostly determined by the water-concentration dependency of *v*. Below the glass transition, PC-SAFT combined with NET-GP predicts a decrease of v. even with increasing water concentration, thus correctly describing the minimum of ${Đ}_{wp}^{\prime \prime }$ in PVP at ~0.11 water weight fraction. In contrast, using PC-SAFT alone always predicts a volume increase upon increasing water concentration, which results in predicted diffusion coefficients ${Đ}_{wp}^{\prime \prime }$ that monotonously increase with increasing water concentration (Figure 5). Thus, the minimum water diffusion coefficients in polymers directly follows the water-induced free-volume effect in polymers correctly described using NET-GP. Decreasing water diffusion coefficients with increasing water concentration below the glass transition were also observed in PVP-water mixtures by Chalykh et al. [7] and in hypromellose acetate succinate-water mixtures by Sturm et al. [44]. They both also explained the decrease in the water diffusion coefficient based on the fractional free volume. For that purpose, Sturm et al. [44] used a modification of the free-volume theory to describe glassy polymer-water mixtures and introduced an additional non-equilibrium contribution that decreases the fractional free volume below the glass transition. Their approach is similar to ours, whereas we use NET-GP to consider the non-equilibrium.

**Figure 6:**
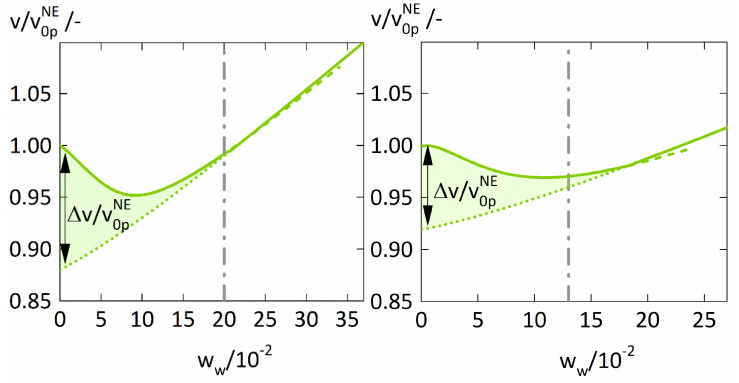
The ratio $\frac{v}{v_{0p}^{NE}}$. of the specific volume v. of the polymer-water mixture relative to the specific volume $v_{0p}^{NE}$ of the dry polymer in non-equilibrium calculated using PC-SAFT alone (dotted lines), PC-SAFT with NET-GP (dashed lines), and the combined approach (thick solid line) for PVP-water (left) and PVPVA-water (right). The quantity $\frac{\Delta v}{v_{0p}^{NE}}\;$. describes the difference between the modelings of $\frac{v}{v_{0p}^{NE}}$. using PC-SAFT alone (dotted lines) and the combined approach (thick solid line) and is highlighted as the filled region. The glass transitions are displayed as dashed lines and were derived from literature [38,40].

Figure 6. shows the specific volumes v. of the polymer-water mixtures relative to the specific volume $v_{0p}^{NE}$ of the dry polymers in non-equilibrium. For both polymers, the ratio $\frac{v}{v_{0p}^{NE}}$ decreases with increasing water concentration, reaches a minimum for water weight fractions of ~0.1, and then rises again. The minima of $\frac{v}{v_{0p}^{NE}}$ directly corresponds to the minima of the Maxwell-Stefan diffusion coefficients ${Đ}_{wp}^{\prime \prime }$. discussed above (Figure 5). Furthermore, $\frac{v}{v_{0p}^{NE}}$ decreases below one for water weight fractions smaller than 0.22, the specific volume *v* of the polymer-water mixture obviously is even smaller than the specific volume $v_{0p}^{NE}$ of the dry (glassy) polymer. This is counterintuitive but was also observed in polyvinylalcohol-water mixtures [45], starch-water mixtures [46], polyamide-water mixtures [47], and polysulfone-solvent systems [48].

This phenomenon is also known as antiplasticization and was already discussed by Mascia et al. [49]. It is known to cause a reduction of the penetrant diffusion coefficient in polymers at increasing penetrant concentration, consequently supporting our results. The antiplasticizing effect of water on PVP and PVPVA is caused by the breakdown of the “frozen” volume in the glassy state, illustrated in Figure 6 as the volume difference $\Delta v=v^{NE}-v^{EQ}$ comparing the volumina in pseudo-equilibrium $v^{NE}$ and in equilibrium $v^{EQ}$. This difference represents the additional free volume available for water diffusion in the glassy polymer and its decrease with increasing water weight fraction consequently decreases the FFV. This adverse effect on the FFV vanishes above the glass transition leaving only the favorable effect of water’s higher free volume on the diffusion coefficient of water in the polymers.

## 5. Conclusions

We measured and modeled water-sorption isotherms and water-sorption kinetics in PVP and PVPVA. Both polymers cross the glass transition upon water uptake. At high RHs, the water-sorption isotherms could be very well described using PC SAFT, whereas significant deviations were observed at low RHs. As these deviations were caused by the polymer-water mixtures being glassy at low RHs, we used PC-SAFT combined with the NET-GP approach to accurately describe also water sorption isotherms at these RHs. As a result, the water-sorption isotherms were satisfactorily described over the entire range of RHs using a combination of PC-SAFT and the NET GP approach. The intersection points of the modeled water-sorption isotherms by PC-SAFT with and without NET GP agreed very well with DSC-measured glass transition temperatures. Additionally, the impact of NET-GP on the modeling of absorption isotherms was smaller for the low-water-absorbing polymer (PVAc) than for the highly-water-absorbing polymers (PVP and PVPVA).

This study provided Fickian diffusion coefficients and Maxwell-Stefan diffusion coefficients of water in PVP and PVPVA over a broad range of relative humidity until 0.9 RH. Anomalous sorption behavior and a concentration dependency of both Fickian diffusion coefficients and Maxwell-Stefan diffusion coefficients were observed. The two water diffusion coefficients were first decreasing with increasing water concentration at lower RHs but then increasing with increasing water concentration at higher RHs. The non-monotonous water concentration dependency of the Maxwell-Stefan was modeled using the free-volume theory. This could explain the resulting minimum in the water diffusion coefficient at medium water concentrations by two counteractive effects on the fractional free volume of the polymer-water mixture when transitioning from the glassy to the rubbery state.

## Figures and Tables

**Table 1:** Mixing rules as used in this work. $k_{ij}$ is the binary interaction parameter between component i and j.

Pure-Component Parameter	Mixing Rule
$\sigma_{i}$	$\sigma_{ij}=0.5\cdot \left(\sigma_{i}+\sigma_{j}\right)$
$u_{i}$	$u_{ij}=\sqrt{u_{i}u_{j}}\left(1-k_{ij}\right)$
$m_{i}$	$\bar{m}=\overset{}{\underset{i}{\sum }}x_{i}m_{i}$
$\varepsilon^{AiBi}$	$\varepsilon^{AiBj}=0.5\cdot \left(\varepsilon^{AiBi}+\varepsilon^{AjBj\;}\right)$
$\kappa^{AiBi}$	$\kappa^{AiBj}=\sqrt{\kappa^{AiBi}\kappa^{AjBj}}{\left(\frac{\sqrt{\sigma_{i}\sigma_{j}}}{\sigma_{ij}}\right)}^{3}$

**Table 2:** PC-SAFT and NET-GP parameters used in this work. $N_{i}$ represents the number of association sites of component i. $\rho_{0i}$ are pure densities of components i taken from literature.

	PVP [26]	PVPVA [27]	PVAc [28]	Water [29]
$M_{i}/\frac{g}{mol}$	25,700	65,000	90,000	18.02
$m_{i}/M_{i}/\frac{mol}{g}$	0.0407	0.0372	0.0321	0.06687
$\sigma_{i}/Å$	2.71	2.947	3.397	2.7971
$u_{i}/k_{B}/\;K$	205.992	205.271	204.650	353.94
$\epsilon^{AiBi}/k_{B}/\;K$	0	0	0	2425.67
$\kappa^{AiBi}/-$	0.02	0.02	0.02	0.0451
$N_{i}/-$	231/231	653/653	1047/1047	1/1
$k_{wi}/-$	−0.128 ^a^	−0.128 ^a^	−0.131 [30]	N.A
$v_{0i}^{NE}/\frac{cm^{3}}{g}\;$	0.6637 ^a^	0.7478 ^a^	0.9174 ^a^	N.A
$k_{wi}^{NE}/\frac{cm^{3}}{g}$	0.4279 ^a^	0.244 ^a^	0	N.A
$\rho_{0i}/\frac{kg}{m^{3}}$	1250	1190	1180	997

a: determined in this study.

**Table 3:** Relative humidity RH, experimental water weight fraction $w_{w}^{\infty }$ at the end of the sorption step, Fickian water diffusion coefficients $D_{wp}$ and Maxwell-Stefan diffusion coefficients ${Đ}_{wp}^{\prime \prime }$ determined in this study at 25 °C.

RH /10^−2^	$w_{wPVP}^{\infty }$ /10^−2^	$w_{wPVPVA}^{\infty }$ /10^−2^	${D_{wPVP}}^{a}$ /10^−15^ m^2^s^−1^	${D_{wPVPVA}}^{b}$ /10^−15^ m^2^s^−1^	${Đ}_{wPVP}^{\prime \prime }$ /10^−15^ m^2^s^−1^	${Đ}_{wPVPVA}^{\prime \prime }$ /10^−15^ m^2^s^−1^
9.24	2.61 ± 0.138	1.1 ± 0.031	88.0 ± 1.99	146.0 ± 30.9	255.4 ± 7.46	340.7 ± 82.4
29.4	7.99 ± 0.134	3.95 ± 0.095	66.7 ± 16.8	94.9 ± 3.74	94.7 ± 27.6	301.5 ± 15.3
44.5	12.6 ± 0.453	6.88 ± 0.12	40.9 ± 5.33	31.3 ± 3.2	57.8 ± 8.34	75.9 ± 8.51
59.9	17.4 ± 0.562	10.6 ± 0.192	65.7 ± 8.25	52.7 ± 6.14	12.4 ± 16.4	106.2 ± 14.9
73.4	23.2 ± 0.692	15.4 ± 0.275	85.0 ± 9.64	85.9 ± 6.34	223.6 ± 23.7	184.3 ± 18.5
87.8	33.0 ± 0.802	24.9 ± 0.43	50.7 ± 4.16	69.8 ± 2.31	345.4 ± 27.7	610.6 ± 39.5

a: the average dry thickness calculated from the dry mass $m_{0}$, density $\rho_{0p}$ and area A of the cylindrical PVP films was 7.61 ± 0.43 µm. b: the average dry thickness calculated from the dry mass $m_{0}$, density $\rho_{0p}$ and area A of the cylindrical PVPVA films was 8.49 ± 0.26 µm.

## Data Availability

Data is contained within the article or supplementary material.

## Supplementary Materials

The following is available online at https://www.mdpi.com/article/10.3390/membranes12040434/s1. Table S1: presenting calculations regarding the sorption rate at the end of each sorption step and the average relative deviations resulting from the fittings to the water sorption kinetics.
