# Supplementary material for: Water Sorption in Glassy Polyvinylpyrrolidone-Based Polymers
Source: Membranes (Basel). 2022 Apr 17;12(4):434. doi: 10.3390/membranes12040434 (PMC9026426; doi:10.3390/membranes12040434)
Supplement: Supplementary file 1 [file membranes-12-00434-s001.zip › membranes-1674557-supplementary.pdf]

membranes

MDPI

Supplementary Material

Water Sorption in Glassy Polyvinylpyrrolidone-based Polymers

Dominik Borrmann [1], Andreas Danzer [1] and Gabriele Sadowski [1,*]

[1] Department of Chemical and Biochemical Engineering, Laboratory of Thermodynamics, TU Dortmund University, Emil-Figge-Str. 70, D-44227 Dortmund, Germany; dominik.borrmann@tu-dortmund.de (D.B.); andreas.danzer@tu-dortmund.de (A.D.)

* Correspondence: Tel.: +49-231-755-2635; gabriele.sadowski@tu-dortmund.de

1. Uncertainty of the water-sorption kinetics' fittings

The uncertainty of the fittings of Equation 9 with the measured water-sorption kinetics of the main manuscript was evaluated by the average relative deviation ARD as displayed in Equation S1.

$$\mathrm{ARD} = \frac{1}{\mathrm{n_t}} \sum_{\mathrm{k}}^{\mathrm{n_t}} \frac{\left|\mathrm{w_{w,k}} - \mathrm{w_{w,k}^{exp}}\right|}{\mathrm{w_{w,k}^{exp}}} \tag{S1}$$

Here, k is the index for the number of data points n_t of the water sorption kinetics, $w_{w,k}^{exp}$ is the measured water weight fraction and $w_{w,k}$ is the modeled water weight fraction determined from Equation 9 of the main manuscript. The ARDs of all water sorption kinetics are displayed in Table S1.

Moreover, the sorption steps of the main manuscript were terminated after 200/120 min assuming a (pseudo-) equilibrium of the polymer-water mixture at the respective relative humidity RH. The validity of this (pseudo-)equilibrium was evaluated via a comparison of an average sorption rate SR^{∞} in this (pseudo-)equilibrium and the initial sorption rate SR^{0}. The sorption rate describes the rate of change of the measured water weight fractions $w_{w,k}^{exp}$. The SR^{∞} was calculated as displayed in Equation S2.

$$\mathrm{SR}^{\infty} = \frac{1}{\mathrm{n_t^*}} \sum_{\mathrm{k=1}}^{\mathrm{n_t-1}} \frac{\mathrm{w_{w,k+1}^{exp}} - \mathrm{w_{w,k}^{exp}}}{\mathrm{t_{k+1}} - \mathrm{t_k}} \ \forall\, \mathrm{k}\ \ \mathrm{t_k} > 60\ \mathrm{min} \tag{S2}$$

n_t^* is the number of data points where the time t_k of the kth data point is greater than 60 min. We estimated that the water sorption kinetics reached a (pseudo-)equilibrium at around 60 min. An average of all sorption rates was taken to prevent a bias resulting from the scattering of $w_{w,k}^{exp}$. The initial sorption rate SR^{0} was calculated as displayed in Equation S3.

$$\mathrm{SR}^{0} = \frac{\mathrm{w_{w,2}^{exp}} - \mathrm{w_{w,1}^{exp}}}{\mathrm{t_2} - \mathrm{t_1}} \tag{S3}$$

Both SR^{∞} and SR^{0} are is also displayed in Table S1.

Table S1. Average relative deviation ARD from the water sorption kinetics' fittings at 25 °C of the main manuscript, average sorption rates in (pseudo-) equilibrium SR^{∞} and initial sorption rates SR^{0} at the respective relative humidity RH.

RH /10^{-2}	ARD_{PVP} /10^{-2}	ARD_{PVPVA} /10^{-2}	SR_{PVP}^{∞} /(µg g^{-1} min^{-1})	SR_{PVPVA}^{∞} /(µg g^{-1} min^{-1})	SR_{PVP}^{0} /(µg g^{-1} min^{-1})	SR_{PVPVA}^{0} /(µg g^{-1} min^{-1})
9.24	10.500 ± 2.292	10.381 ± 1.484	0.132 ± 0.179	0.017 ± 0.024	63.39 ± 2.45	30.01 ± 4.88

29.4	2.816 ± 0.475	2.813 ± 0.191	0.212 ± 0.137	0.073 ± 0.066	104.86 ± 35.37	86.67 ± 12.20
44.5	0.845 ± 0.117	1.108 ± 0.107	0.169 ± 0.102	0.166 ± 0.026	72.89 ± 11.33	41.90 ± 5.216
59.9	1.018 ± 0.067	1.033 ± 0.046	0.165 ± 0.281	< 0.001	88.65 ± 31.16	47.29 ± 8.42
73.4	0.705 ± 0.054	1.047 ± 0.031	0.126 ± 0.191	< 0.001	103.02 ± 13.92	79.87 ± 11.49
87.8	0.517 ± 0.057	0.597 ± 0.048	0.028 ± 0.351	0.08 ± 0.456	204.96 ± 49.37	240.28 ± 13.01

The ARDs decrease from 10% to 0.5% with increasing RH, supporting that anomalous sorption behavior occurs mostly in the glassy polymer-water mixtures. The very high ARD of 10% for the lowest RH step limits the significance of the determined water diffusion coefficient in the main manuscript.

All average sorption rates in (pseudo-) equilibrium SR^{∞} were lower than 0.212 μg g^{-1} min^{-1}. In contrast, the initial sorption rate SR^{0}(with 30–240 μg g^{-1} min^{-1}) is several magnitudes more significant than the SR^{∞} , showing that the polymer-water mixtures were in a (pseudo-)equilibrium after 60 min.
